# The Metastatic Potential and Chemoresistance of Human Pancreatic Cancer Stem Cells

**DOI:** 10.1371/journal.pone.0148807

**Published:** 2016-02-09

**Authors:** Vikash J. Bhagwandin, J. Michael Bishop, Woodring E. Wright, Jerry W. Shay

**Affiliations:** 1 Department of Cell Biology, University of Texas Southwestern Medical Center, Dallas, Texas; 2 G.W. Hooper Research Foundation, University of California San Francisco, San Francisco, California, United States of America; University of Nebraska Medical Center, UNITED STATES

## Abstract

Cancer stem cells (CSCs) typically have the capacity to evade chemotherapy and may be the principal source of metastases. CSCs for human pancreatic ductal carcinoma (PDAC) have been identified, but neither the metastatic potential nor the chemoresistance of these cells has been adequately evaluated. We have addressed these issues by examining side-population (SP) cells isolated from the Panc-1 and BxPC3 lines of human PDAC cells, the oncogenotypes of which differ. SP cells could be isolated from monolayers of Panc-1, but only from spheroids of BxPC3. Using orthotopic xenografts into the severely immunocompromised NSG mouse, we found that SP cells isolated from both cell lines produced tumors that were highly metastatic, in contrast to previous experience with PDAC cell lines. SP cells derived from both cell lines expressed the ABCG2 transporter, which was demonstrably responsible for the SP phenotype. SP cells gave rise to non-SP (NSP) cells *in vitro* and *in vivo*, a transition that was apparently due to posttranslational inhibition of the ABCG2 transporter. Twenty-two other lines of PDAC cells also expressed ABCG2. The sensitivity of PDAC SP cells to the vinca alkaloid vincristine could be greatly increased by verapamil, a general inhibitor of transporters. In contrast, verapamil had no effect on the killing of PDAC cells by gemcitabine, the current first-line therapeutic for PDAC. We conclude that the isolation of SP cells can be a convenient and effective tool for the study of PDAC CSCs; that CSCs may be the principal progenitors of metastasis by human PDAC; that the ABCG2 transporter is responsible for the SP phenotype in human PDAC cells, and may be a ubiquitous source of drug-resistance in PDAC, but does not confer resistance to gemcitabine; and that inhibition of ABCG2 might offer a useful adjunct in a therapeutic attack on the CSCs of PDAC.

## Introduction

Pancreatic ductal adenocarcinoma (PDAC) ranks among the most lethal of human malignancies. Despite aggressive surgery and use of contemporary neoadjuvant and adjuvant chemotherapy, the overall 5-year survival is approximately 10–26% depending on the severity of dissemination (cancer.org and cancer.net). Chemotherapeutic agents such as gemcitabine reduce primary tumor burden, but fail to eliminate metastatic liver disease, the major cause of mortality in PDAC. The cancer stem cell hypothesis suggests that a small population of stem-like cells is responsible for the persistent growth of a tumor and resistance to chemotherapeutics [1]. In addition, it has been proposed that CSCs may be the immediate source of metastatic cells that intrinsically withstand chemotherapy [1,2]. Several groups have isolated and characterized pancreatic CSCs by using combinations of cell surface markers [either CD44^+^/CD24^+^/ESA^+^ [3], or CD133^+^/CXCR4^+^ [4]], or by SP fractionation [5–8]. The CSCs expressing CD44^+^/CD24^+^/ESA^+^ obtained from patient samples or CD133^+^/CXCR4^+^ from cell lines have been shown to be highly tumorigenic in NOD/SCID mice [3,4]. However, the metastatic potential of tumors derived from CD44^+^/CD24^+^/ESA^+^ CSCs has not been reported, and the tumors elicited with CD133^+^/CXCR4^+^ CSCs were only weakly metastatic[4]. Alternatively, the procedure of SP fractionation has been used to enrich for pancreatic CSCs, but tumors initiated by these CSCs also metastasized poorly [5,6]. We have used orthotopic injection into severely immunodeficient mice to improve the evaluation of SP cells derived from PDAC cell lines. Our results suggest that the CSCs included in PDAC SP fractions may be the progenitors of metastasis, and demonstrate that the transporter ABCG2 is a widespread, if not ubiquitous source of potential drug resistance in PDAC, but is not responsible for resistance to the first-line therapeutic gemcitabine. SP cells derived from human PDAC cell lines offer a convenient means by which to explore the mechanisms of tumorigenesis, metastasis and drug resistance.

## Materials and Methods

### Cell Lines

Pancreatic cancer cell lines were obtained from ATCC (Manassas, VA) and grown in DMEM for Panc-1 cells, or RPMI for BxPC3 cells supplemented with 10% FBS. The BxPC3 cell line was also grown as spheroids, using uncoated bacteriological petri dishes (non-TC) and serum-free DMEM supplemented with B27 (Invitrogen), a proprietary reagent that is known to promote the growth of CD133^+^/CXCR4^+^ CSCs [9]. The adrenal cortical carcinoma cell line H295 was grown in DMEM supplemented with insulin, transferrin and selenium (ITS), and 10% FBS.

### *In Vitro* Pharmacology

For dose response studies with gemcitabine, 1500 SP cells from Panc-1 or 10000 SP cells from BxPC3 were plated into 96-well dishes and treated 24 hours later with two-fold serial dilutions of gemcitabine with or without 20μM of verapamil. For vincristine studies, Panc-1, BxPC3, or H295 SP cells were plated into 96-well dishes. Each cell line was treated with two-fold serial dilutions of vincristine with or without 50μM of verapamil. After one week, cells were stained using the MTT assay. In each experiment, the BxPC3 cells were grown as spheroids on non-TC 96-well dishes in serum-free DMEM supplemented with B27.

### MTT Assay

The MTT assay was used to determine the degree of chemoresistance in SP and NSP cells. The media from drug treated cells was replaced with 100μl of MTT substrate (5μg/ml) diluted in assay media (phenol-free DMEM, 25mM HEPES, 1mM Na-Pyruvate) and placed in a tissue culture incubator for 4 hours. The substrate was replaced with 100μl of solubilization solution (10% Triton X-100, 0.1N HCl, 80% Isopropanol) and gently shaken for 5 minutes. The plates were read in a Tecan2 plate reader at a detection wavelength of 570nm, and reference of 690nm.

### Immunostaining of ABC transporters

Panc-1, BxPC3 or H295 cells were trypsinized, washed two times with PBS, fixed with 0.1% PFA for 10 min and permeabilized with 0.3% saponin in FACS buffer. Both cell types were stained with BXP-53 (ABCG2, Santa Cruz) or G-1 (ABCB1/MDR-1, Santa Cruz) antibody diluted 1:100 in FACS buffer for 30 min on ice, washed twice with PBS, stained with FITC 1:1000 in FACS buffer for 30 min on ice, washed with PBS, and analyzed on a FACSaria. Immunohistochemistry: 5μM sections were cut from paraffin embedded tissues of primary tumors, deparaffinized with xylenes, hydrated through graded alcohols to PBS. The sections were subjected to heat induced epitope retrieval, and residual peroxidase activity was quenched with PBS/3% hydrogen peroxide mix. Staining was performed using the Vectastain ABC elite Rabbit IgG kit (cat# PK-6101, Vectorlabs) with ABCG2 primary antibody 1:100 dilution overnight (cat# GTX100436, Genetex) and counterstained with Meyer’s Hematoxylin.

### Side population assay

SP assays were performed as previously reported [7,10]. Briefly, 1 x 10^6^ cells were stained with Hoechst 33342 (HOE) at a final concentration of 5μg/ml and verapamil controls were pre-treated with 100μM of verapamil for 10min. All samples were incubated at 37°C degrees for 60 min with intermittent mixing every 15 min. Cells were collected and resuspended in PBS with 3% BSA, 0.01% DNase I, and 1μg/ml of propidium iodide and filtered through a 40μM cell strainer. The BxPC3 spheroids were dissociated by normal trypsinization prior to HOE staining. Immuno-inhibition assay: prior to HOE staining, Panc-1, BxPC3 and H295 cells were pre-treated with 5μg of either ABCG2 antibody, 5D3 (R&D systems, cat# MAB995) or MDR1 antibody, MRK-16 (Kamiya Biomedical, cat# MC-017).

### Orthotopic Xenograft

This study was conducted in strict accordance with the recommendations in the Guide for the Care and Use of Laboratory Animals of the National Institutes of Health. The protocol was approved by the University of California at San Francisco’s Animal Use Committee (protocol number: AN090185-01). For orthotopic xenografts, mice were anesthetized with ketamine and xylazine. Mice were sacrificed by injection with ketamine and xylazine, followed by exsanguination. The NOD-SCID/IL-2 gamma (NSG) mice were purchased from Jackson laboratories for all *in vivo* procedures. Mice were anesthetized by intraperitoneal injection of a 50μl cocktail containing, 25mg/ml ketamine, 2.5mg/ml xylazine, and 0.5mg/ml acepromazine. After mice were anesthetized the site of incision was sterilized with betadine and cleaned with an alcohol swab. A small incision was made below the costal margin and the pancreas was exposed using forceps without damaging or disconnecting the organ and placed onto the exterior of the abdomen. The pancreas was held with gentle traction and a suspension of cells in 50μl of matrigel was injected into the tail under the capsule of the pancreas. A sterile cotton-tip swab was applied to the site of injection for 5 seconds to allow the pancreatic capsule to reseal. The injected pancreas was then returned to the peritoneal cavity through the initial incision. The site of incision was sutured and treated with topical antibiotic cream. Finally, the animal was placed in a warm cage and injected with post surgical analgesics as necessary.

### Luciferase tagging of PDAC cells and *In vivo* Bioluminescence Imaging

A four-plasmid transfection system was used to make lentivirus. Plasmids obtained from Geron Corp. encoding tet-rev, gag-pol, vsv-g, and pMND-luc were transfected into HEK293 cell line using Fugene 6 following vendor transfection recommendations. The cells were recovered after 24 hours and viral supernatants were collected every 12 hours for 72 hours total. Supernatants were filtered using a 0.45mM filter and mixed DEAD-dextran at 4 μg/ml and added to Panc-1 or BxPC3 cells for transduction. After 72 hours of sequential infections, the cells were recovered in normal culture media for three days. The cells were then split at a 1:10 ratio and placed under G418 selection at 1mg/ml for 10 days. Live cells were collected and lysed using Promega’s luciferase lysis buffer system for 30 minutes on ice and then measured on a luminometer. *In vivo* bioluminescence imaging was conducted on a Xenogen imaging apparatus using conditions previously described [11].

## Results

### The SP phenotype in monolayers and spheroid cultures of human PDAC cell lines

**Fig 1:**
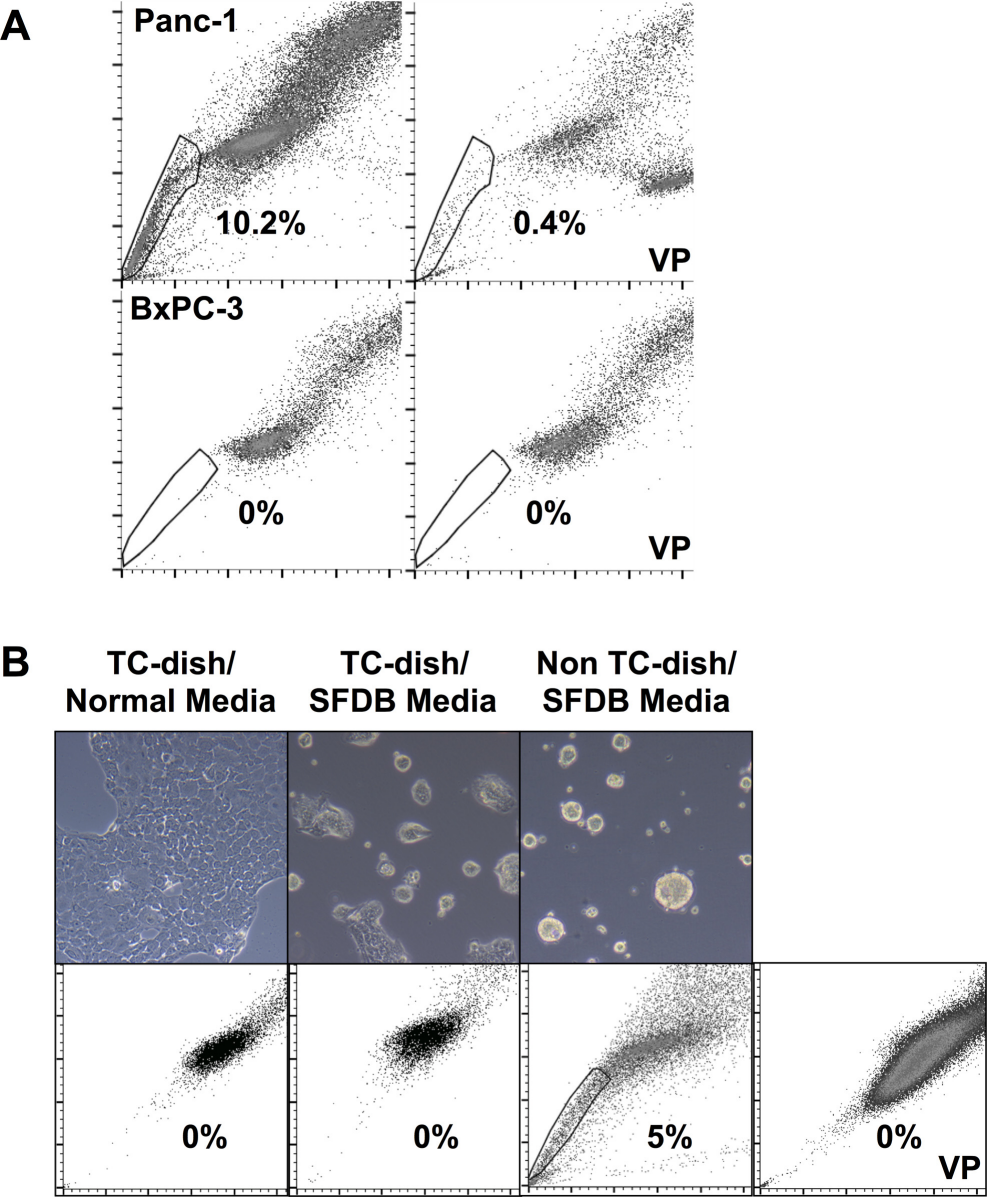
The SP phenotype in monolayers and spheroid cultures of human PDAC cell lines. (A) Panc-1 and BxPC3 cells were stained with Hoechst 33342 dye (5μg/ml) and analyzed with a FACSaria flow cytometer, as described in the Materials and Methods. The gate for SP cells was determined by treatment with verapamil (100μM). The fraction of cells in the SP gate is given in each panel. Profile of Panc-1 cells, upper row; and BxPC3 cells, lower row; verapamil control (VP), right panels. (B) Top row: BxPC3 cells were grown either in standard medium on tissue culture dishes (TC), in serum-free DMEM with B27 (SFDB) on TC dishes, or as spheroids in SFDB on uncoated bacteriological (non-TC) petri dishes. Bottom row: quantification of SP by FACS sorting for each culture condition, verapamil control (VP), far right panel.

We first compared the SP content of two human PDAC cell lines, the oncogenotypes of which differ: Panc-1 cells, which contain an oncogenic *KRAS* mutation and a loss of function *TP53* mutation (both of which are common in PDAC); and BxPC3 cells, which contain a loss of function mutation in *SMAD4* and *CDKN2A* genes (both of which are relatively uncommon in PDAC) [12]. The SP fraction was determined by detecting the exclusion of the DNA binding dye Hoechst 33342 (HOE) from cells, an indication of transporter activity [4,10]. The gate for SP was determined by verapamil inhibition, a broadly active transporter antagonist. A 10% SP fraction was detected in Panc-1 cells (Fig 1A), but contrary to a previous report [5], an SP fraction could not be detected in BxPC3 cells propagated in conventional medium as 2D monolayers (Fig 1A). The SP fraction previously reported in BxPC3 cells was not tested in a biological assay and therefore may be an issue of flow cytometry instrument type, instrument settings or staining method. In an effort to uncover CSCs in the BxPC3 cell line, unfractionated cells were grown as spheroids under conditions designed to foster the survival and propagation of normal stem cells (see Materials and Methods). This produced an SP fraction of 5% in the cultures of BxPC3 spheroids (Fig 1B). In contrast, Panc-1 cells failed to thrive when cultured under the conditions used with BxPC3 and could not be used for SP fractionation. The ability of spheroids to reveal the SP phenotype in BxPC3 is consistent with previous reports that CSCs are enriched in spheroids [13–15]. It is notable that the two PDAC cell lines have distinctly different oncogenotypes, which may account for the differences in their SP content and response to the specialized medium.

### Tumor initiating potential of SP and NSP cells

**Fig 2:**
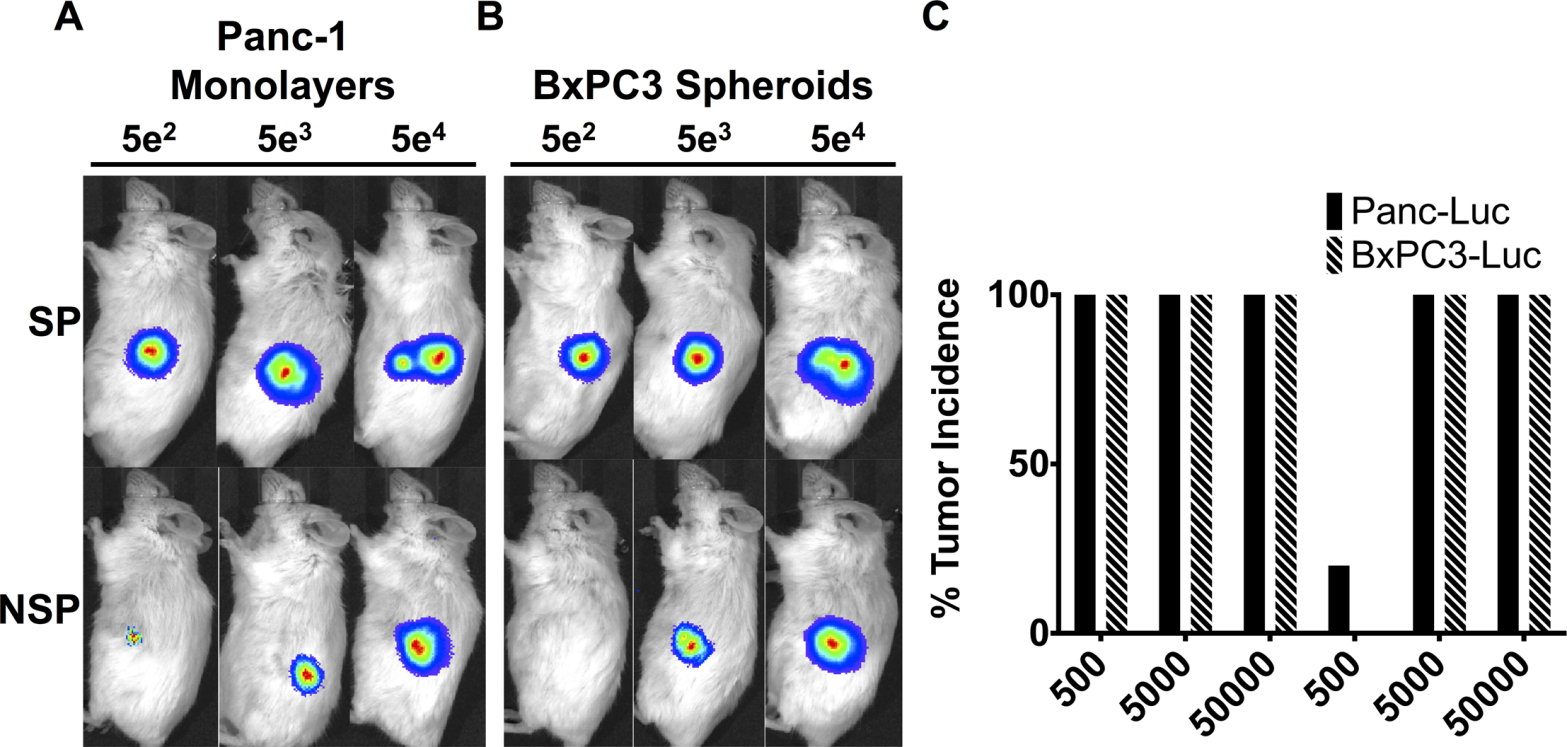
Tumor initiating potential of SP and NSP cells. SP and NSP cells were obtained by fractionation using the gates shown in Fig 1. The tumor initiating potential of SP and NSP was tested by orthotopically injecting 500, 5000, or 50000 cells of luciferase-labeled cells from each population, using cohorts of five NSG mice for each dose of cells. After two weeks, the relative amount of tumor volume was detected using real-time *in vivo* bioluminescence imaging. (A) Panc-1 monolayer cells (B) BxPC3 spheroids cells. (C) Histogram of tumor incidence for each cohort of five NSG mice, Student’s *t* test = *P*<0.002.

**Fig 3:**
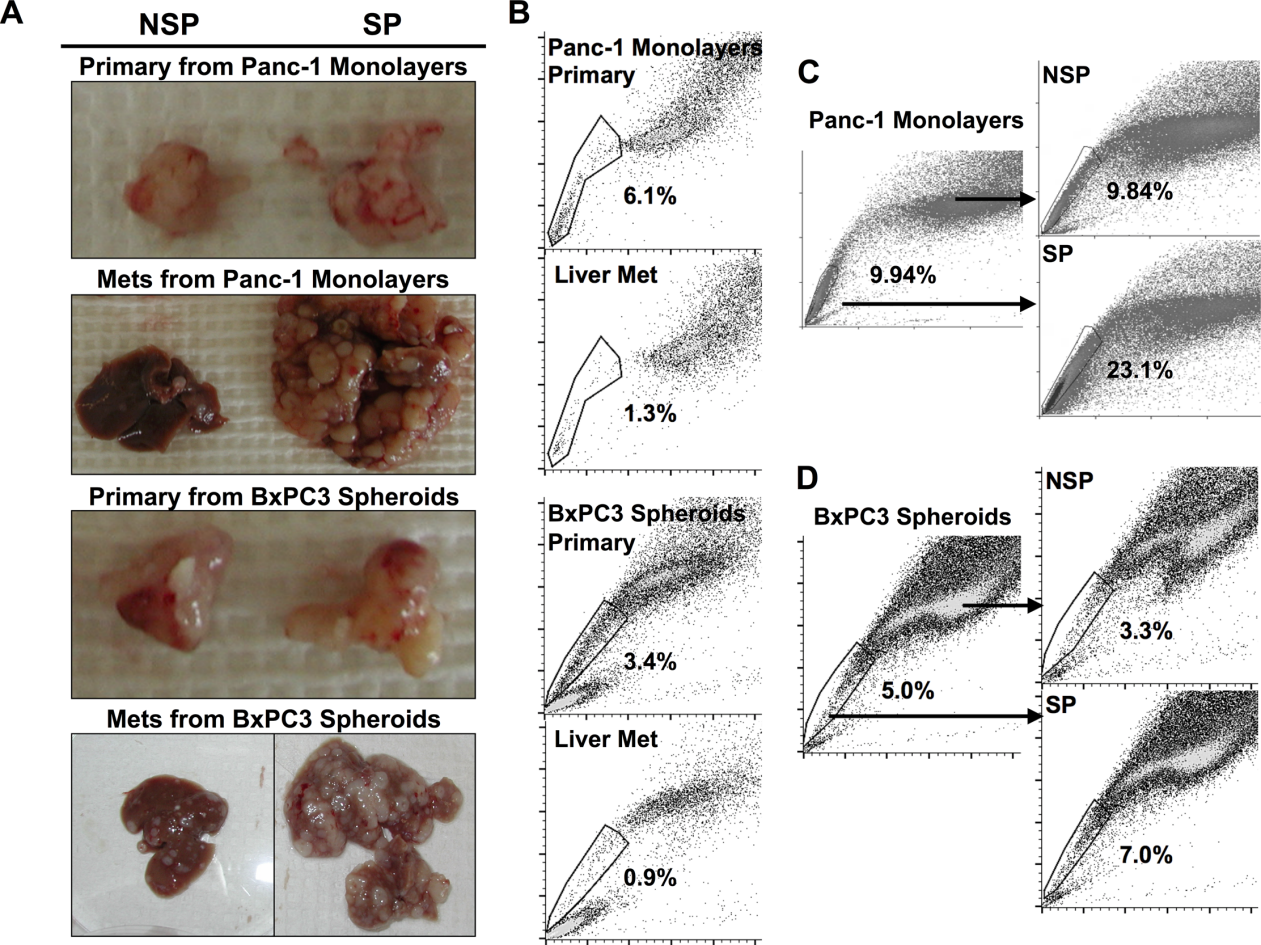
Metastatic potential of SP CSCs. SP and NSP cells were isolated from Panc-1 monolayers and BxPC3 spheroids, then orthotopically injected as 500 cell aliquots into cohorts of three NSG mice. The animals were maintained for three months and then examined for primary and metastatic tumors. (A) Comparison of primary tumors and metastases generated by SP, right column and NSP cells, left column isolated from Panc-1 monolayers and metastases isolated from BxPC3 spheroids. The upper row illustrates primary tumors and the second row illustrates metastases for both cell lines. (B) Tumor cells were isolated from primary and metastatic tumors and analyzed for SP content. Primary tumor site, top panels; metastatic site, bottom panels. (C-D) SP and NSP fractions were isolated from Panc-1 and BxPC3 cells and plated back into culture, using standard medium for cells from Panc-1 on normal tissue culture dishes and spheroid medium for BxPC3 cells on non-TC dishes. After one week without passage, each plated fraction was analyzed by FACS. The percentage of cells in the SP gate is given in each panel.

Based on our interpretation of the published data [3,5,6,8], CSCs enriched by selection for cell surface markers are substantially more efficient than SP CSCs in tumorigenesis assays. In an effort to improve the performance of SP CSCs in assays for tumorigenesis, we utilized orthotopic injection into the pancreata of NSG mice, whose severe immunodeficiency makes them exceptionally sensitive to tumorigenesis by xenografts [16]. We orthotopically injected 500, 5000, or 50000 of SP or NSP cells fractionated from luciferase-tagged Panc-1 monolayers or BxPC3 spheroids. The luciferase was used to obtain images from injected mice. We found that 500 SP cells were sufficient to produce tumors within two weeks in the entire cohort of five mice, whereas 500 NSP cells did so in only a single mouse out of five (Fig 2A and 2B). By three months, however, all of the animals had developed pancreatic tumors (see below, Fig 3A). These results are a decided improvement upon previous experience with SP cells, in which a minimum of 10,000 cells was required to initiate tumorigenesis that was detectable within 4–5 weeks [5,6]. We conclude that our protocol has improved the efficiency of tumorigenesis by CSCs contained in the SP fraction from PDAC cell lines.

### Metastatic potential of SP CSCs

Recent evidence suggests that CSCs may be the source of metastatic disease [17,18]. However, previous efforts failed to obtain vigorous metastasis with CSCs enriched by the use of either cell surface markers or SP fractionation. This was true of cells representing either PDAC or other forms of malignancy [3,4,19–24]. In an effort to augment metastasis by tumors formed from SP cells, we again used orthotopic injection into NSG mice. We isolated SP and NSP fractions from Panc-1 monolayer cells and BxPC3 spheroid cells, then injected 500 of these cells into the pancreata of three NSG mice in each cohort. By three months, both SP and NSP cells from each cell line had produced primary tumors of approximately 1cm in size (Fig 3A). In addition, there was severe metastatic liver disease in the mice injected with SP cells, virtually effacing the normal liver parenchyma (Fig 3A, right panels). The animals that had received NSP cells also contained hepatic metastases, but the metastases were both few in number and significantly smaller (Fig 3A, left panels). We conclude that tumors produced with SP cells derived from either monolayers or spheroids are aggressively metastatic.

Primary tumors initiated with SP cells from either Panc-1 or BxPC3 contained an SP fraction of roughly the same size as that found in the cell lines (Fig 3B). Moreover, the size of the SP fraction was even lower in the metastases (Fig 3B), in agreement with previous reports [8,25]. These results suggest that CSCs or other components of the SP fraction population spawned numerous NSP cells during the course of tumorigenesis and metastasis. Further evidence of such an event was observed when SP and NSP cells were fractionated from Panc-1 monolayers and BxPC3 spheroids, and then cultivated *in vitro* without passage. After one week, with only 2.5 doublings, a large majority of the cultured SP cells from both lines now fractionated as NSP cells (Fig 3C and 3D), thus seeming to replicate the events that occurred *in vivo* during the course of tumorigenesis. In contrast, the cultured NSP cells from both lines contained modest quantities of SP cells (Fig 3C and 3D). This finding could represent either a trace contamination of the NSP fraction or a low level of conversion of NSP cells to the SP phenotype. Whatever their source, the presence of SP cells in the NSP fraction represents a potential explanation for the tumor initiated by the NSP fraction in Fig 2A, the apparent ability of NSP cells to produce tumors in a delayed manner (Fig 3A), and the metastases that occurred in mice that received NSP cells (Fig 3A).

### ABCG2 is responsible for the SP phenotype in PDAC cell lines

**Fig 4:**
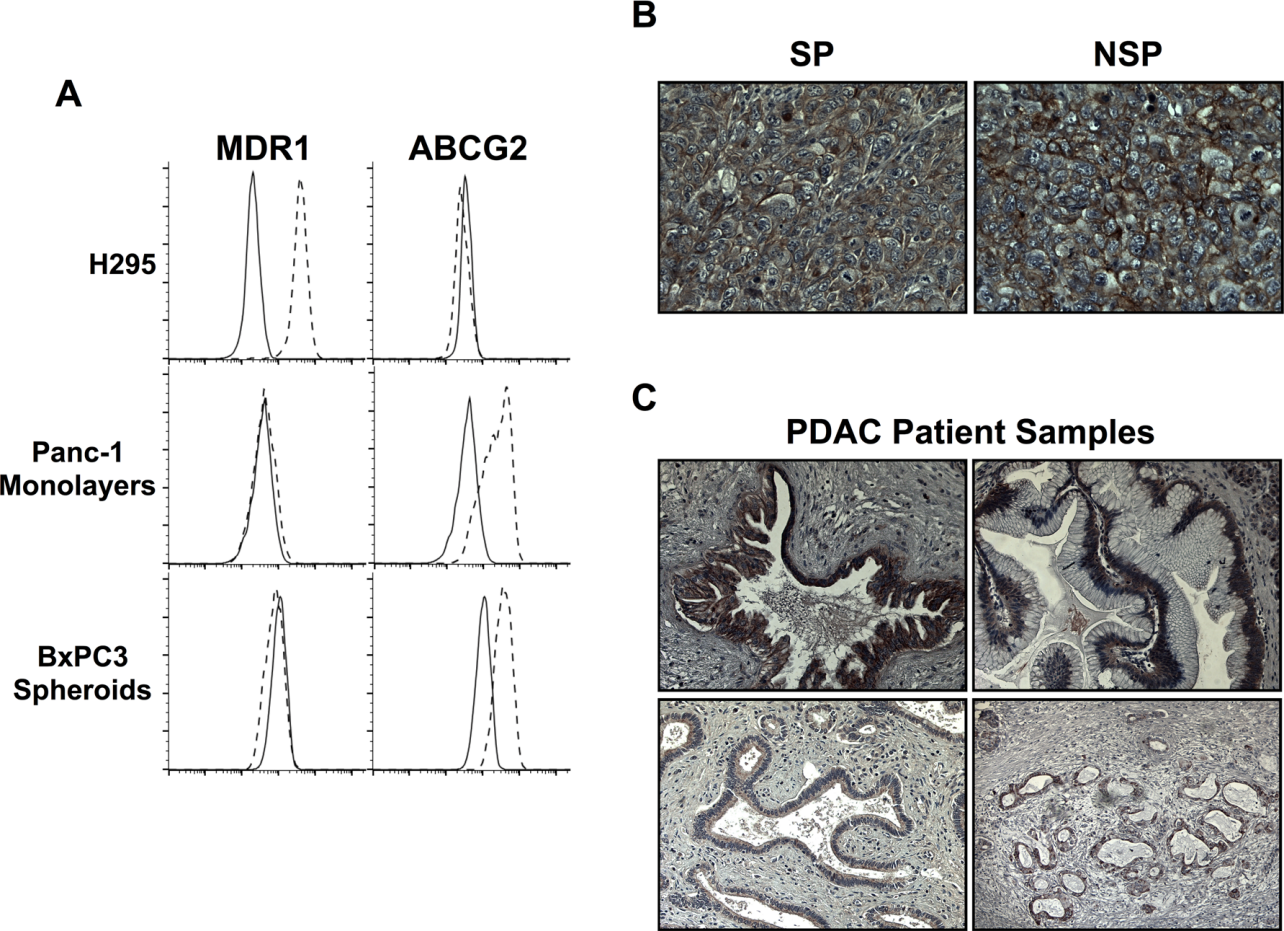
ABCG2 expression in PDAC cell lines and primary tumors. (A) Cell surface staining of ABCG2 (5D3 antibody) and ABCB1/MDR-1 (MRK-16 antibody) on Panc-1 monolayer cells, BxPC3 spheroid cells and H295 cells. (B) Immunohistochemistry staining of ABCG2 on SP and NSP generated primary tumors (positive DAB stain, brown). (C) Immunohistochemistry staining of ABCG2 on primary PDAC tumors from four patients (positive DAB stain, brown).

The ATP binding cassette (ABC) transporters are an important source of drug resistance in human tumors [26,27]. Two of these transporters, ABCG2/BCRP and ABCB1/MDR-1, are reported to be the principal sources of the SP phenotype in human tumor cell lines [19,20]. It has been reported previously that PDAC cells express ABCG2, but not MDR-1 [5,6,28,29]. However, ABCG2 activity is dependent on expression at the cell surface, which is regulated by posttranslational modification and compartmentalization [30]. Therefore, we stained for the two transporters on the surface of Panc-1 and BxPC3 cells. As a control, we used H295 cells, adrenocortical carcinoma cells that express MDR-1 [31,32]. We found that Panc-1 and BxPC3 cells expressed only ABCG2 on their surface, whereas H295 cells expressed only MDR-1 (Fig 4A). The FACS data show that the entire population of Panc-1 and BxPC3 cells expressed ABCG2 (Fig 4A), and we obtained similar results by using immunohistochemistry to examine primary tumors generated from SP and NSP cells, and four human PDAC patient samples (Fig 4B and 4C).

**Fig 5:**
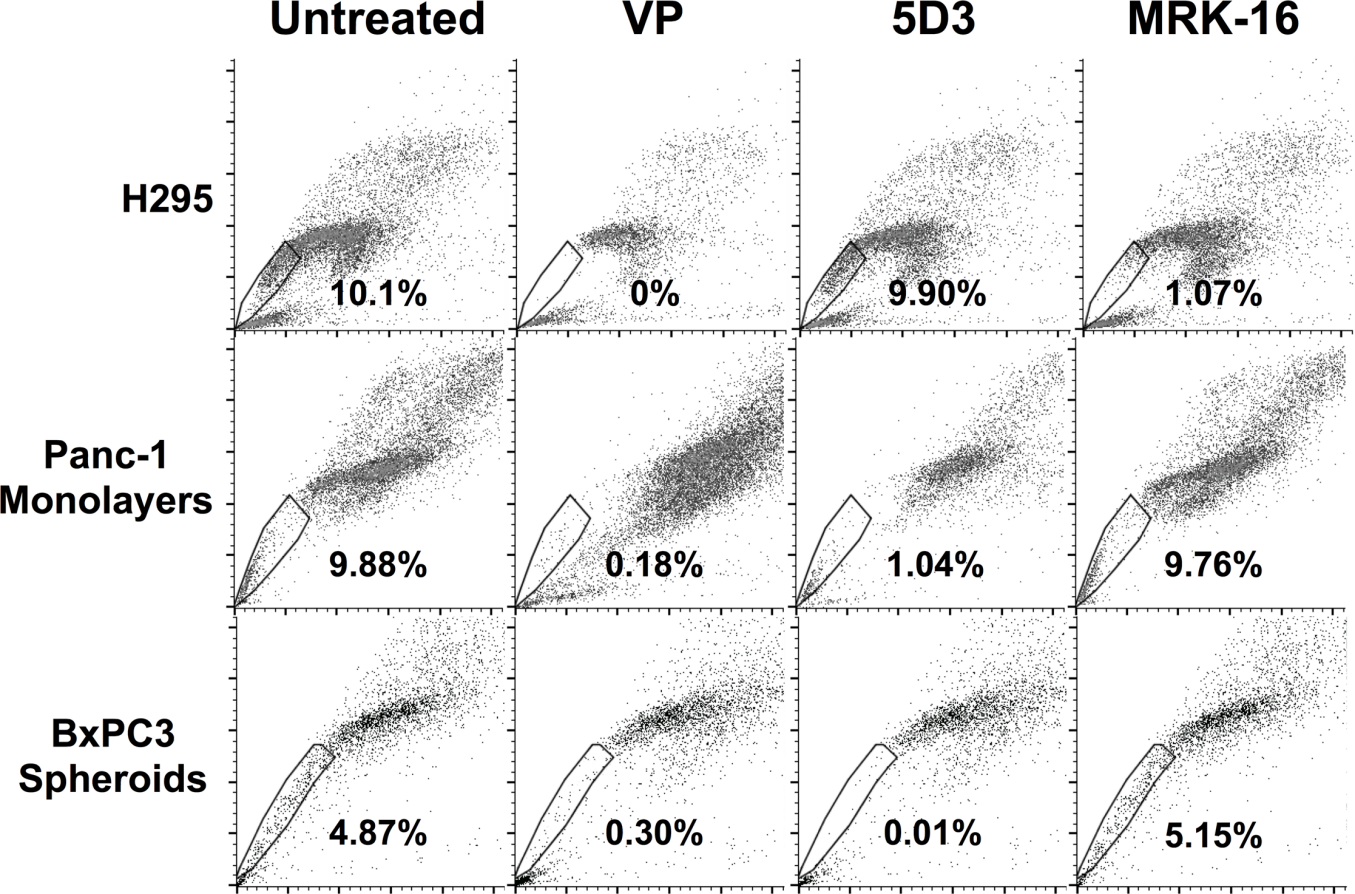
Characterization of ABCG2 activity in PDAC cell lines. Inhibition of the SP phenotype. Cells were treated with blocking antibodies for ABCG2 (5D3) and MDR-1 (MRK-16), and then fractionated by FACS, as described in the Materials and Methods. H295, top row; Panc-1 monolayer, middle row; and BxPC3 spheroids, bottom row. Columns 1–4 are as labeled in the figure: untreated, verapamil (VP), 5D3 antibody, and MRK-16 antibody.

The ubiquity of ABCG2 in the cell populations raised the unorthodox possibility that the transporter was not responsible for the SP phenotype. In order to test this possibility, we treated cells with blocking antibodies for ABCG2 [33] and MDR-1 [34] prior to performing the SP fractionation. Inhibition of ABCG2 reduced the SP fraction of Panc-1 cells and BxPC3 spheroids to 1.04% (9.5 fold reduction) and 0.01% (400 fold reduction), respectively (Fig 5). In contrast, inhibition of MDR-1 had no effect on the SP content of the two PDAC cell lines, but reduced the SP fraction in H295 cells by more than nine-fold (Fig 5). We conclude that the ABCG2 transporter is responsible for the SP phenotype in Panc-1 and BxPC3 cells. Thus, despite the ubiquity of the transporter in the cell populations, it must be active in only the limited portion of the cells that sort as SP. It has been reported previously that the activity of ABCG2 is subject to post-translational control by Akt [30], which might account for the existence of NSP cells.

**Fig 6:**
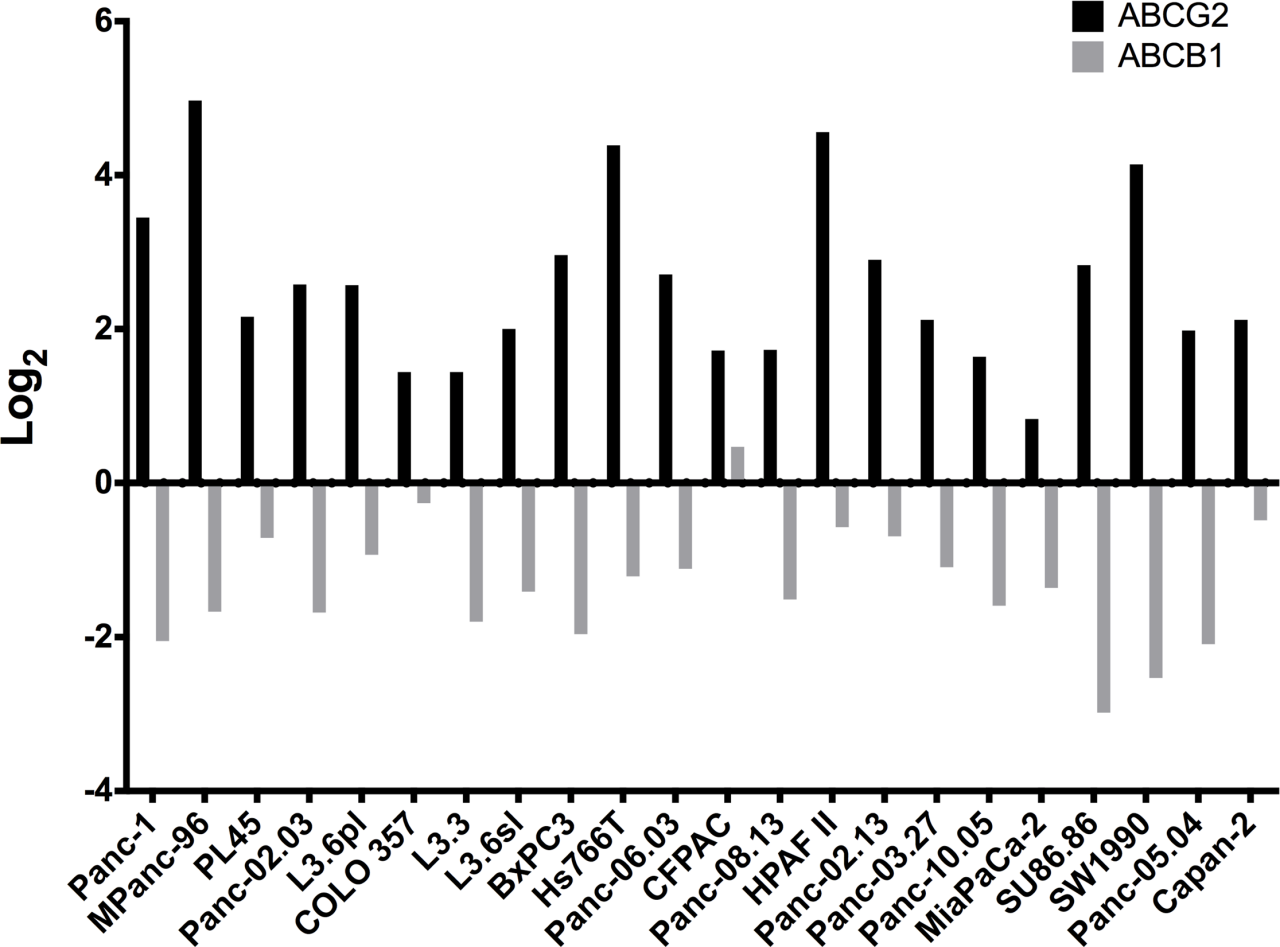
Comparison of ABCG2 and MDR-1 expression in PDAC cell lines. Microarray analysis of ABCG2 and MDR-1 in 22 pancreatic cancer cell lines [36]. The log_2_ expression data for ABCG2 and MDR-1 from three non-malignant human pancreatic ductal epithelial cell lines (hPDEC; #904, #916, #515) were averaged and subtracted from the log_2_ from each pancreatic cancer cell line and plotted as a Waterfall and Manhattan plot, Student’s *t* test = *P*<0.001.

In order to determine how widespread expression of ABCG2 might be in PDAC, we obtained previously published microarray data from 22 pancreatic cancer cell lines [35] and compared the expression of ABCG2 to that of MDR-1. Three normal pancreatic epithelial cell lines were also analyzed in the array study and were used here to normalize the cancer cell line data. Strikingly, every pancreatic cancer cell line expressed ABCG2 by 2–5 fold above normal pancreatic epithelial cell lines, whereas none of the cells expressed MDR-1 (Fig 6). We conclude that expression of ABCG2 may be a ubiquitous property of PDAC cells, perhaps representing an atavism from a normal stem-like cell that gives rise to CSCs, and thus, to tumors.

### The ABCG2 transporter does not confer resistance to gemcitabine

**Fig 7:**
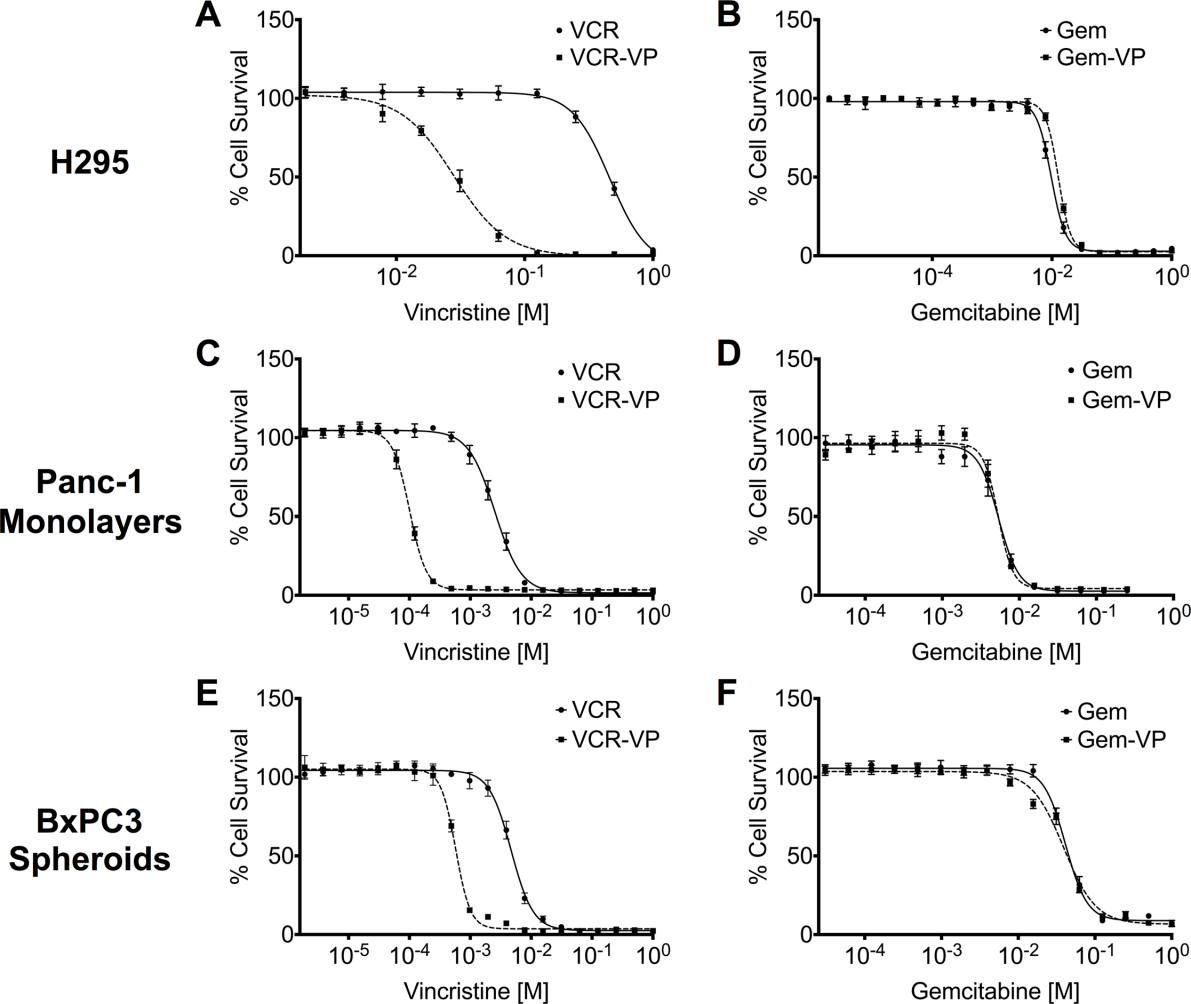
The ABCG2 transporter does not confer resistance to Gemcitabine. The dose response of SP cells from (A) H295, (C) Panc-1 monolayer cells, and (E) BxPC3 spheroid cells to vincristine (VCR) and combination of vincristine with verapamil (VCR-VP) was determined using the MTT assay. Similarly, the dose response of SP cells from (B) H295, (D) Panc-1 monolayer cells and (E) BxPC3 spheroid cells to gemcitabine (Gem) and the combination of gemcitabine with verapamil (Gem-VP) was determined using the MTT assay. All data points were generated from a single experiment and represent the mean of six wells per treatment concentration, normalized to the mean of six internal untreated control wells, then transformed by natural log and displayed as a four parameter variable plot as a function of log (drug) versus response. Error bars represent standard error of mean (SEM) from six wells in a single experiment, all of the graphs were generated using Prism statistical analysis software, Student’s *t* test = *P*<0.0002.

Gemcitabine is generally the first-line treatment for patients with PDAC, but the dismal survival rate for the disease reflects a relatively limited response to the drug. The resistance of PDAC SP cells to gemcitabine has been attributed to efflux by ABCG2 [5]. If that were correct, then inhibition of the transporter by the broadly active antagonist verapamil should increase the sensitivity of PDAC cells to gemcitabine [27]. We first confirmed the presence of transporter-mediated resistance in the PDAC cell lines by treating SP cells from Panc-1, BxPC3 and H295 with either vincristine or a combination of vincristine and verapamil (Fig 7A, 7C and 7E and Table 1). Verapamil greatly enhanced the killing by vincristine of SP cells from all three lines. In contrast, verapamil had no effect on the killing of Panc-1 and BxPC3 SP cells by gemcitabine (Fig 7B, 7D and 7E and Table 1), indicating that the presence of ABCG2 or MDR-1 had not conferred resistance to gemcitabine on either cell line. We conclude that the transporter normally resident in PDAC cells does not contribute to the limitations of gemcitabine as a therapeutic for the disease.

**Table 1:** Sensitivity of SP cells chemotherapeutics.

Cell Type	Gem	Gem + VP	Fold Change
**H295**	9.68 nM + 3.78	12.7 nM + 3.69	0
**Panc-1 Monolayers**	5.35 nM + 1.34	5.31 nM + 1.23	0
**BxPC3 Spheroids**	41.3 nM + 0.93	39.2 nM + 1.31	0
	**VCR**	**VCR + VP**	
**H295**	466 nM + 3.95	31.4 nM + 2.28	15
**Panc-1 Monolayers**	2.66 nM + 0.97	0.10 nM + 0.49	27
**BxPC3 Spheroids**	4.69 + 0.72	0.58 nM + 0.70	8

IC_50_ for SP cells from H295, Panc-1 monolayers and BxPC3 spheroids when treated with gemcitabine (Gem), vincristine (VCR) or the combination of VCR with verapamil (VP). The IC_50_ was determined by the MTT assay and expressed in nM. All values were derived from the four parameter variable plot obtained in Fig 7 and displayed with standard error of mean (SEM) using Prism statistical analysis software.

## Discussion

### The SP Cells of PDAC

Established lines of human cancer cells have long been used as experimental surrogates for samples taken directly from tumors, for both the study of tumorigenesis and screening for new therapeutics. The utility of cell lines derived from human PDAC, however, has been compromised by the failure of tumors produced with such cells to display the robust metastasis that is so typical of the clinical disease. Here we demonstrate a solution to this problem. When orthotopically injected into severely immunocompromised NSG mice, SP cells from PDAC cell lines gave rise to tumors that were aggressively metastatic. In contrast, NSP cells were less tumorigenic, and the resulting tumors were poorly metastatic. Since the SP fraction is enriched for CSCs, these findings conform to the view that CSCs may be the principal progenitors for metastasis [2,17,18]. Neoplastic organoids derived from both murine and human PDAC also give rise to metastatic disease when grafted orthotopically into immunocompromised mice [16]. It remains to be seen whether the metastases in this system originate from a distinctive subset of cells in the tumors, and if so, whether they are the CSCs of the tumor.

A recent report has described separation of CSCs from the SP fraction of several tumor types by the use of autofluorescence [15]. In contrast, the SP fraction that we derived from PDAC cell lines displayed an enriched ability to produce highly metastatic tumors, properties consistent with the presence of CSCs. We note that there was a substantial difference in how the two studies prepared cells for FACS sorting. Otherwise, we cannot explain the discrepancy between the two sets of results.

In order to obtain an SP fraction from the BxPC3 cell line, we had to propagate the cells under conditions that fostered growth as spheroids. We can think of two possible explanations for this observation. First, SP cells may be present in the line, but in numbers too low for detection by SP fractionation. Since the growth conditions were designed to favor stem-like cells, they might provide a selective advantage for the survival and propagation of SP cells, including the subset of these cells that behave as CSCs in tumorigenesis assays. It is notable that spheroids are reputed to be inherently enriched for CSCs [9,13,14]. Alternatively, the cell culture medium might promote conversion of NSP to SP cells, similar to the conversion from Non-CSCs to CSC described previously for human melanoma and breast cancer [37,38]. We gained a hint of such conversion from the presence of SP cells in cultures of NSP cells, although the SP cells might have arisen instead from a selective advantage provided to contaminating SP cells by propagation *in vitro*. Whatever the explanation for the behavior of BxPC3 cells, the results suggest that cultivation of cells as spheroids might be a general means to uncover an SP phenotype in tumor cell lines that otherwise do not display that phenotype.

Previous reports have described the ability of SP cells to spawn NSP cells [21–25,39–41], much as CSCs serve as the source for the more mature cells that compose the bulk of tumors. In accord with these observations, there was an abundance of NSP cells in the PDAC tumors initiated by SP cells in our experiments, and cultivating SP cells *in vitro* resulted in NSP cells quickly becoming the large majority of the population. Since the entire population of cells contains ABCG2, a possible explanation for the transition to the NSP phenotype is the previously described post-translational inhibition of transporter activity by Akt [30].

### The ABCG2 transporter as mediator of the SP phenotype and chemoresistance

We identified the ABCG2 transporter as the mediator of the SP phenotype in Panc-1 and BxPC3 cells, and found expression of that transporter in twenty-two other PDAC cell lines. The apparent ubiquity of ABCG2 in PDAC cells suggests that expression of the transporter might be a reversion to an evolutionary ancestral type similar to a normal stem or progenitor cell that gives rise to the stem cell for PDAC. It remains unclear to what extent ABCG2 is responsible for the refractory nature of PDAC. On the one hand, we observed that ABCG2 does not efflux gemcitabine, a lead drug in the treatment of PDAC. Instead, resistance of PDAC to gemcitabine is apparently caused by other factors, such as activation of two anti-apoptotic genes, BCL2 and BCL-XL [42–44], or a deficit in the transmembrane importer for gemcitabine [45–50]. On the other hand, ABCG2 does efflux vincristine, a component of standard CHOP therapy, and we found that inhibition of ABCG2 with verapamil greatly sensitized PDAC SP cells to vincristine. Verapamil is too toxic to be useful in a clinical setting, but our results suggest that more tractable inhibitors of ABCG2 might be valuable adjuncts in the treatment of PDAC.

## References

[1] Reya T, Morrison SJ, Clarke MF, Weissman IL. Stem cells, cancer, and cancer stem cells. Nature. 2001;414: 105–111. 10.1038/35102167 11689955

[2] Pardal R, Clarke MF, Morrison SJ. Applying the principles of stem-cell biology to cancer. Nat Rev Cancer. 2003;3: 895–902. 10.1038/nrc1232 14737120

[3] Li C, Heidt DG, Dalerba P, Burant CF, Zhang L, Adsay V, et al Identification of pancreatic cancer stem cells. Cancer Res. 2007;67: 1030–1037. 10.1158/0008-5472.CAN-06-2030 17283135

[4] Hermann PC, Huber SL, Herrler T, Aicher A, Ellwart JW, Guba M, et al Distinct populations of cancer stem cells determine tumor growth and metastatic activity in human pancreatic cancer. Cell Stem Cell. 2007;1: 313–323. 10.1016/j.stem.2007.06.002 18371365

[5] Yao J, Cai H-H, Wei J-S, An Y, Ji Z-L, Lu Z-P, et al Side population in the pancreatic cancer cell lines SW1990 and CFPAC-1 is enriched with cancer stem-like cells. Oncol Rep. 2010;23: 1375–1382. 20372854 10.3892/or_00000774

[6] Wang YH, Li F, Luo B, Wang XH, Sun HC, Liu S, et al A side population of cells from a human pancreatic carcinoma cell line harbors cancer stem cell characteristics. Neoplasma. 2009;56: 371–378. 19580337 10.4149/neo_2009_05_371

[7] Bhagwandin VJ, Shay JW. Pancreatic cancer stem cells: fact or fiction? Biochim Biophys Acta. 2009;1792: 248–259. 10.1016/j.bbadis.2009.02.007 19233264 PMC2670354

[8] Kabashima A, Higuchi H, Takaishi H, Matsuzaki Y, Suzuki S, Izumiya M, et al Side population of pancreatic cancer cells predominates in TGF-beta-mediated epithelial to mesenchymal transition and invasion. Int J Cancer. 2009;124: 2771–2779. 10.1002/ijc.24349 19296540

[9] Lonardo E, Hermann PC, Mueller M-T, Huber S, Balic A, Miranda-Lorenzo I, et al Nodal/Activin signaling drives self-renewal and tumorigenicity of pancreatic cancer stem cells and provides a target for combined drug therapy. Cell Stem Cell. 2011;9: 433–446. 10.1016/j.stem.2011.10.001 22056140

[10] Goodell MA, Brose K, Paradis G, Conner AS, Mulligan RC. Isolation and functional properties of murine hematopoietic stem cells that are replicating in vivo. J Exp Med. 1996;183: 1797–1806. 8666936 10.1084/jem.183.4.1797 PMC2192511

[11] Sennino B, Ishiguro-Oonuma T, Wei Y, Naylor RM, Williamson CW, Bhagwandin V, et al Suppression of tumor invasion and metastasis by concurrent inhibition of c-Met and VEGF signaling in pancreatic neuroendocrine tumors. Cancer Discovery. 2012;2: 270–287. 10.1158/2159-8290.CD-11-0240 22585997 PMC3354652

[12] Sipos B, Möser S, Kalthoff H, Török V, Löhr M, Klöppel G. A comprehensive characterization of pancreatic ductal carcinoma cell lines: towards the establishment of an in vitro research platform. Virchows Arch. 2003;442: 444–452. 10.1007/s00428-003-0784-4 12692724

[13] Yin T, Wei H, Gou S, Shi P, Yang Z, Zhao G, et al Cancer stem-like cells enriched in panc-1 spheres possess increased migration ability and resistance to gemcitabine. Int J Mol Sci. 2011;12: 1595–1604. 10.3390/ijms12031595 21673909 PMC3111620

[14] Du Z, Qin R, Wei C, Wang M, Shi C, Tian R, et al Pancreatic cancer cells resistant to chemoradiotherapy rich in “stem-cell-like” tumor cells. Dig Dis Sci. 2011;56: 741–750. 10.1007/s10620-010-1340-0 20683663

[15] Miranda-Lorenzo I, Dorado J, Lonardo E, Alcala S, Serrano AG, Clausell-Tormos J, et al Intracellular autofluorescence: a biomarker for epithelial cancer stem cells. Nat Meth. 2014;11: 1161–1169. 10.1038/nmeth.3112 25262208

[16] Quintana E, Shackleton M, Sabel MS, Fullen DR, Johnson TM, Morrison SJ. Efficient tumour formation by single human melanoma cells. Nature. 2008;456: 593–598. 10.1038/nature07567 19052619 PMC2597380

[17] Oskarsson T, Batlle E, Massagué J. Metastatic Stem Cells: Sources, Niches, and Vital Pathways. Cell Stem Cell. Elsevier Inc; 2014;14: 306–321. 10.1016/j.stem.2014.02.002 24607405 PMC3998185

[18] Malanchi I, Santamaria-Martínez A, Susanto E, Peng H, Lehr H-A, Delaloye J-F, et al Interactions between cancer stem cells and their niche govern metastatic colonization. Nature. Nature Publishing Group; 2013;481: 85–89. 10.1038/nature10694 22158103

[19] Kondo T, Setoguchi T, Taga T. Persistence of a small subpopulation of cancer stem-like cells in the C6 glioma cell line. Proc Natl Acad Sci USA. 2004;101: 781–786. 10.1073/pnas.0307618100 14711994 PMC321758

[20] Patrawala L, Calhoun T, Schneider-Broussard R, Zhou J, Claypool K, Tang DG. Side population is enriched in tumorigenic, stem-like cancer cells, whereas ABCG2+ and ABCG2- cancer cells are similarly tumorigenic. Cancer Res. 2005;65: 6207–6219. 10.1158/0008-5472.CAN-05-0592 16024622

[21] Szotek PP, Pieretti-Vanmarcke R, Masiakos PT, Dinulescu DM, Connolly D, Foster R, et al Ovarian cancer side population defines cells with stem cell-like characteristics and Mullerian Inhibiting Substance responsiveness. Proc Natl Acad Sci USA. 2006;103: 11154–11159. 10.1073/pnas.0603672103 16849428 PMC1544057

[22] Wang J, Guo L-P, Chen L-Z, Zeng Y-X, Lu SH. Identification of cancer stem cell-like side population cells in human nasopharyngeal carcinoma cell line. Cancer Res. 2007;67: 3716–3724. 10.1158/0008-5472.CAN-06-4343 17440084

[23] Ho MM, Ng AV, Lam S, Hung JY. Side population in human lung cancer cell lines and tumors is enriched with stem-like cancer cells. Cancer Res. 2007;67: 4827–4833. 10.1158/0008-5472.CAN-06-3557 17510412

[24] Wu C, Wei Q, Utomo V, Nadesan P, Whetstone H, Kandel R, et al Side population cells isolated from mesenchymal neoplasms have tumor initiating potential. Cancer Res. 2007;67: 8216–8222. 10.1158/0008-5472.CAN-07-0999 17804735

[25] Yin L, Castagnino P, Assoian RK. ABCG2 expression and side population abundance regulated by a transforming growth factor beta-directed epithelial-mesenchymal transition. Cancer Res. 2008;68: 800–807. 10.1158/0008-5472.CAN-07-2545 18245481

[26] Shen DW, Fojo A, Chin JE, Roninson IB, Richert N, Pastan I, et al Human multidrug-resistant cell lines: increased mdr1 expression can precede gene amplification. Science. 1986;232: 643–645. 3457471 10.1126/science.3457471

[27] Gottesman MM, Pastan I. Biochemistry of multidrug resistance mediated by the multidrug transporter. Annu Rev Biochem. 1993;62: 385–427. 10.1146/annurev.bi.62.070193.002125 8102521

[28] Wang F, Xue X, Wei J, An Y, Yao J, Cai H, et al hsa-miR-520h downregulates ABCG2 in pancreatic cancer cells to inhibit migration, invasion, and side populations. Br J Cancer. 2010;103: 567–574. 10.1038/sj.bjc.6605724 20628378 PMC2939772

[29] Zhou J, Wang C-Y, Liu T, Wu B, Zhou F, Xiong J-X, et al Persistence of side population cells with high drug efflux capacity in pancreatic cancer. World J Gastroenterol. 2008;14: 925–930. 18240351 10.3748/wjg.14.925 PMC2687061

[30] Takada T, Suzuki H, Gotoh Y, Sugiyama Y. Regulation of the cell surface expression of human BCRP/ABCG2 by the phosphorylation state of Akt in polarized cells. Drug Metab Dispos. 2005;33: 905–909. 10.1124/dmd.104.003228 15843490

[31] Bates SE, Shieh CY, Mickley LA, Dichek HL, Gazdar A, Loriaux DL, et al Mitotane enhances cytotoxicity of chemotherapy in cell lines expressing a multidrug resistance gene (mdr-1/P-glycoprotein) which is also expressed by adrenocortical carcinomas. J Clin Endocrinol Metab. 1991;73: 18–29. 1675220 10.1210/jcem-73-1-18

[32] Bello-Reuss E, Ernest S, Holland OB, Hellmich MR. Role of multidrug resistance P-glycoprotein in the secretion of aldosterone by human adrenal NCI-H295 cells. Am J Physiol, Cell Physiol. 2000;278: C1256–65. 10837354 10.1152/ajpcell.2000.278.6.C1256

[33] Nakanishi T, Shiozawa K, Hassel BA, Ross DD. Complex interaction of BCRP/ABCG2 and imatinib in BCR-ABL-expressing cells: BCRP-mediated resistance to imatinib is attenuated by imatinib-induced reduction of BCRP expression. Blood. 2006;108: 678–684. 10.1182/blood-2005-10-4020 16543472

[34] Mechetner EB, Roninson IB. Efficient inhibition of P-glycoprotein-mediated multidrug resistance with a monoclonal antibody. Proc Natl Acad Sci USA. 1992;89: 5824–5828. 1352877 10.1073/pnas.89.13.5824 PMC402110

[35] Gysin S, Rickert P, Kastury K, McMahon M. Analysis of genomic DNA alterations and mRNA expression patterns in a panel of human pancreatic cancer cell lines. Genes Chromosomes Cancer. 2005;44: 37–51. 10.1002/gcc.20216 15929091

[36] Gysin S, Paquette J, McMahon M. Analysis of mRNA Profiles after MEK1/2 Inhibition in Human Pancreatic Cancer Cell Lines Reveals Pathways Involved in Drug Sensitivity. Mol Cancer Res. 2012;10: 1607–1619. 10.1158/1541-7786.MCR-12-0188 22833572 PMC4261949

[37] Quintana E, Shackleton M, Foster HR, Fullen DR, Sabel MS, Johnson TM, et al Phenotypic heterogeneity among tumorigenic melanoma cells from patients that is reversible and not hierarchically organized. Cancer Cell. 2010;18: 510–523. 10.1016/j.ccr.2010.10.012 21075313 PMC3031091

[38] Iliopoulos D, Hirsch HA, Wang G, Struhl K. Inducible formation of breast cancer stem cells and their dynamic equilibrium with non-stem cancer cells via IL6 secretion. Proc Natl Acad Sci USA. 2011;108: 1397–1402. 10.1073/pnas.1018898108 21220315 PMC3029760

[39] Chow EK-H, Fan L-L, Chen X, Bishop JM. Oncogene-specific formation of chemoresistant murine hepatic cancer stem cells. Hepatology. 2012 10.1002/hep.25776 PMC3418440 22505225

[40] Mitsutake N, Iwao A, Nagai K, Namba H, Ohtsuru A, Saenko V, et al Characterization of side population in thyroid cancer cell lines: cancer stem-like cells are enriched partly but not exclusively. Endocrinology. 2007;148: 1797–1803. 10.1210/en.2006-1553 17234707

[41] Hirschmann-Jax C, Foster AE, Wulf GG, Nuchtern JG, Jax TW, Gobel U, et al A distinct “side population” of cells with high drug efflux capacity in human tumor cells. Proc Natl Acad Sci USA. 2004;101: 14228–14233. 10.1073/pnas.0400067101 15381773 PMC521140

[42] Shi X, Liu S, Kleeff J, Friess H, Büchler MW. Acquired resistance of pancreatic cancer cells towards 5-Fluorouracil and gemcitabine is associated with altered expression of apoptosis-regulating genes. Oncology. 2002;62: 354–362. 12138244 10.1159/000065068

[43] Xu Z, Friess H, Solioz M, Aebi S, Korc M, Kleeff J, et al Bcl-x(L) antisense oligonucleotides induce apoptosis and increase sensitivity of pancreatic cancer cells to gemcitabine. Int J Cancer. 2001;94: 268–274. 11668508 10.1002/ijc.1447

[44] Bold RJ, Chandra J, McConkey DJ. Gemcitabine-induced programmed cell death (apoptosis) of human pancreatic carcinoma is determined by Bcl-2 content. Ann Surg Oncol. 1999;6: 279–285. 10340887 10.1007/s10434-999-0279-x

[45] Okazaki T, Javle M, Tanaka M, Abbruzzese JL, Li D. Single nucleotide polymorphisms of gemcitabine metabolic genes and pancreatic cancer survival and drug toxicity. Clin Cancer Res. 2010;16: 320–329. 10.1158/1078-0432.CCR-09-1555 20028759 PMC2802655

[46] Giovannetti E, Mey V, Nannizzi S, Pasqualetti G, Del Tacca M, Danesi R. Pharmacogenetics of anticancer drug sensitivity in pancreatic cancer. Mol Cancer Ther. 2006;5: 1387–1395. 10.1158/1535-7163.MCT-06-0004 16818496

[47] Giovannetti E, Danesi R, Mey V, Nannizzi S, Pasqualetti G, Del Tacca M. In vitro studies on gemcitabine combinations with other antiblastics. Ann Oncol. 2006;17 Suppl 5: v17–19. 10.1093/annonc/mdj943 16807450

[48] Giovannetti E, Del Tacca M, Mey V, Funel N, Nannizzi S, Ricci S, et al Transcription analysis of human equilibrative nucleoside transporter-1 predicts survival in pancreas cancer patients treated with gemcitabine. Cancer Res. 2006;66: 3928–3935. 10.1158/0008-5472.CAN-05-4203 16585222

[49] Spratlin J, Sangha R, Glubrecht D, Dabbagh L, Young JD, Dumontet C, et al The absence of human equilibrative nucleoside transporter 1 is associated with reduced survival in patients with gemcitabine-treated pancreas adenocarcinoma. Clin Cancer Res. 2004;10: 6956–6961. 10.1158/1078-0432.CCR-04-0224 15501974

[50] Griffiths M, Beaumont N, Yao SY, Sundaram M, Boumah CE, Davies A, et al Cloning of a human nucleoside transporter implicated in the cellular uptake of adenosine and chemotherapeutic drugs. Nat Med. 1997;3: 89–93. 8986748 10.1038/nm0197-89

